# Protein-Bound Uremic Toxins in Hemodialysis Patients Relate to Residual Kidney Function, Are Not Influenced by Convective Transport, and Do Not Relate to Outcome

**DOI:** 10.3390/toxins12040234

**Published:** 2020-04-07

**Authors:** Maaike K. van Gelder, Igor R. Middel, Robin W. M. Vernooij, Michiel L. Bots, Marianne C. Verhaar, Rosalinde Masereeuw, Muriel P. Grooteman, Menso J. Nubé, M. A. van den Dorpel, Peter J. Blankestijn, Maarten B. Rookmaaker, Karin G.F. Gerritsen

**Affiliations:** 1 Department of Nephrology and Hypertension, University Medical Center Utrecht, 3584 CX Utrecht, The Netherlands; m.k.vangelder-5@umcutrecht.nl (M.K.v.G.); R.W.M.Vernooij-2@umcutrecht.nl (R.W.M.V.); M.C.Verhaar@umcutrecht.nl (M.C.V.); P.J.Blankestijn@umcutrecht.nl (P.J.B.); M.Rookmaaker@umcutrecht.nl (M.B.R.); 2 Division of Pharmacology, Utrecht Institute for Pharmaceutical Sciences, Utrecht University, 3584 CG Utrecht, The Netherlands; i.r.middel@students.uu.nl (I.R.M.); R.Masereeuw@uu.nl (R.M.); 3 Julius Center for Health Sciences and Primary Care, University Utrecht, 3584 CG Utrecht, The Netherlands; M.L.Bots@umcutrecht.nl; 4 Amsterdam UMC, Vrije Universiteit Amsterdam, Amsterdam Cardiovascular Sciences, Nephrology, 1081 HV Amsterdam, The Netherlands; mpc.grooteman@amsterdamumc.nl (M.P.G.); mensonube@gmail.com (M.J.N.); 5 Department of Internal Medicine, Maasstad Hospital, 3079 DZ Rotterdam, The Netherlands; DorpelM@maasstadziekenhuis.nl

**Keywords:** End-stage kidney disease, Protein-bound uremic toxin, hemodialysis, hemodiafiltration, convection, residual kidney function

## Abstract

Protein-bound uremic toxins (PBUTs) are predominantly excreted by renal tubular secretion and hardly removed by traditional hemodialysis (HD). Accumulation of PBUTs is proposed to contribute to the increased morbidity and mortality of patients with end-stage kidney disease (ESKD). Preserved PBUT excretion in patients with residual kidney function (RKF) and/or increased PBUT clearance with improved dialysis techniques might improve the prognosis of patients with ESKD. The aims of this study are to explore determinants of PBUTs in HD patients, and investigate whether hemodiafiltration (HDF) lowers PBUT plasma concentrations, and whether PBUTs are related to the outcome. Predialysis total plasma concentrations of kynurenine, kynurenic acid, indoxyl sulfate, indole-3-acetic acid, p-cresyl sulfate, p-cresyl glucuronide, and hippuric acid were measured by UHPLC-MS at baseline and after 6 months of follow-up in the first 80 patients participating in the CONvective TRAnsport Study (CONTRAST), a randomized controlled trial that compared the effects of online HDF versus low-flux HD on all-cause mortality and new cardiovascular events. RKF was inversely related to kynurenic acid (*p* < 0.001), indoxyl sulfate (*p* = 0.001), indole-3-acetic acid (*p* = 0.024), p-cresyl glucuronide (*p* = 0.004) and hippuric acid (*p* < 0.001) plasma concentrations. Only indoxyl sulfate decreased by 8.0% (−15.3 to 34.6) in patients treated with HDF and increased by 11.9% (−15.4 to 31.9) in HD patients after 6 months of follow-up (HDF vs. HD: *p* = 0.045). No independent associations were found between PBUT plasma concentrations and either risk of all-cause mortality or new cardiovascular events. In summary, in the current population, RKF is an important determinant of PBUT plasma concentrations in HD patients. The addition of convective transport did not consistently decrease PBUT plasma concentrations and no relation was found between PBUTs and cardiovascular endpoints.

## 1. Introduction

The kidney has two mechanisms to excrete uremic toxins into the urine. Small water-soluble solutes (e.g., urea) are primarily eliminated by glomerular filtration and protein-bound uremic toxins (PBUTs) primarily by tubular secretion mediated by transporters, including organic anion transporters (OAT) and organic cation transporters (OCT) for removal of PBUTs [1,2,3]. Hemodialysis (HD) is a life-sustaining renal replacement therapy that partially replaces renal glomerular filtration, but not renal tubular function. The latter may contribute to the high morbidity and mortality in dialysis patients.

With conventional HD, small (<500 Da) water-soluble molecules are efficiently removed by diffusion. However, PBUTs are difficult to remove by conventional dialysis because of their strong protein-binding. Despite increases in dialysis dose, as measured by small hydrophilic solute clearance (Kt/V urea) [4,5,6], outcome remains poor. Increasing evidence suggests that accumulation of toxins that are not cleared by conventional HD, i.e., PBUTs, may contribute to increased risk of cardiovascular disease. It is of note that residual kidney function (RKF) has been associated with improved survival in dialysis patients, possibly via preserved PBUT clearance [7,8,9]. These findings drawn attention to PBUTs as a potential target for removal by renal replacement therapy.

Several specific PBUTS have been shown to be associated with cardiovascular disease, both in vitro and *in vivo*. Preclinical studies demonstrated that indoxyl sulfate and p-cresyl sulfate, two of the most widely studied PBUTs, play a role in the pathophysiology of cardiovascular disease (CVD). *In vitro*, both toxins have been shown to induce oxidative stress in endothelial cells and inhibit endothelial proliferation and wound repair, and indoxyl sulfate was shown to stimulate the proliferation of vascular smooth muscle cells [10]. Exposure of rats to indoxyl sulfate and p-cresyl sulfate induced vascular calcification in the aorta and peripheral arteries via activation of inflammation and coagulation [11]. In patients with chronic kidney disease (CKD), elevated levels of indoxyl sulfate and p-cresyl sulfate are associated with increased risk of all-cause mortality, and p-cresyl sulfate was associated with an increased risk of cardiovascular events [12]. Administration of the oral sorbent AST-120, that prevents intestinal absorption of PBUT precursors, has been associated with a lower risk of mortality and cardiovascular events [13,14].

Hemodiafiltration (HDF) combines diffusive and convective transport (i.e., high volume ultrafiltration) to enhance removal of middle molecules. A meta-analysis of four randomized controlled trials, comparing the effects of HDF and HD, showed that HDF reduced the risk of all-cause and cardiovascular mortality in dialysis patients, especially at higher convection volumes [15]. It has been shown that HDF may enhance PBUT removal per session [16], but it is unknown whether HDF lowers PBUT plasma concentrations during prolonged treatment and whether this contributes to the improved outcome. Only small studies with limited follow-up duration have compared PBUT removal in patients treated with HDF and HD and reported results were inconsistent [17,18,19,20,21,22].

The first aim of the present study was to explore determinants of total PBUT plasma concentrations in HD patients, including RKF. The second aim was to compare the change in PBUT plasma concentrations in patients treated with HDF and low-flux HD after six months of treatment. Third, associations between PBUT plasma concentrations and all-cause mortality and cardiovascular events were studied.

## 2. Results

### 2.1. Baseline Characteristics

Baseline characteristics of the study population (*n* = 80) are presented in Table 1. The mean age was 62.9 ± 14.2 years, and 56% were male. RKF was present in 53% of the participants, and the median residual creatinine clearance in these patients was 3.6 (1.6 to 7.2) mL/min. Baseline kynurenic acid and indoxyl sulfate plasma concentrations were higher (both *p* < 0.05) in the HD treatment arm, while other patient characteristics, including the number of patients with RKF and renal creatinine clearance, were balanced between both treatment groups.

### 2.2. Determinants of PBUT Plasma Concentrations at Baseline

**Figure 1:**
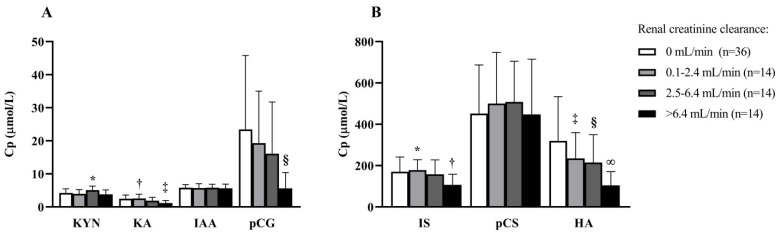
Protein-bound uremic plasma concentrations stratified for renal creatinine clearance categories at baseline. Cp, plasma concentration; HA, hippuric acid; IAA, indole-3-acetic acid; IS, indoxyl sulfate; KA, kynurenic acid; KYN, kynurenine; pCG, p-cresyl glucuronide; pCS, p-cresyl sulfate. One-way ANOVA with Tukey’s honestly significant difference post hoc test. The natural logarithm of hippuric acid, p-cresyl sulfate, p-cresyl glucuronide and indole-3-acetic acid was used for analysis. (**A**): * *p* = 0.036 vs. 2.5-6.4 mL/min; † *p* = 0.002 vs. 0 mL/min; ‡ *p* = 0.007 vs. 0 mL/min; § *p* = 0.022 vs. 0 mL/min. (**B**): * *p* = 0.023 vs. 2.5-6.4 mL/min; † *p* = 0.013 vs. 0 mL/min; ‡ *p* = 0.003 vs. 6.4 mL/min; § *p* = 0.007 vs. 6.4 mL/min; ∞ *p* < 0.001 vs. 0 mL/min.

The association between PBUT plasma concentrations and various patient characteristics is presented in Table 2. Renal creatinine clearance was inversely related to most measured PBUTs: kynurenic acid, indoxyl sulfate, indole-3-acetic acid, p-cresyl glucuronide, and hippuric acid. Stratification according to renal creatinine clearance categories, to better illustrate the inverse relation between PBUT plasma concentration and renal creatinine clearance, showed that kynurenic acid, indoxyl sulfate, and hippuric acid plasma concentrations were higher in patients without or with relatively low residual renal creatinine clearance (0.1–2.4 mL/min) compared with patients with a relatively high creatinine clearance (>6.5 mL/min) (Figure 1). In addition, a relationship between PBUT plasma concentrations and dietary protein intake was observed for two PBUTs—normalized protein nitrogen appearance (nPNA) was directly related to kynurenic acid and indoxyl sulfate. No consistent relationships between PBUT plasma concentrations and dialysis vintage, medications, plasma albumin, blood pressure, history of CVD, diabetes mellitus, and inflammation were observed, as well as not after adjustment for protein intake (nPNA, Table S2) or stratification for RKF (Table S3).

### 2.3. PBUT Change over Time, HDF versus HD

In the HDF group, the mean delivered convection volume was 17.3 ± 4.3 L per session. The percentage change in PBUT plasma concentration from baseline to 6 months of follow-up in the HD and HDF treatment arms and the difference in the rate of change between HD and HDF is presented in Table 3. The percentage change in plasma concentrations from baseline to 6 months of follow-up was only different for indoxyl sulfate between the HD and HDF treatment arms. Indoxyl sulfate plasma concentrations tended to decrease with 8.0% (IQR −15.3 to 34.6) per 6 months in patients treated with HDF, whereas plasma concentrations tended to increase in patients treated with low-flux HD with 11.9% (−15.4 to 31.9) (HDF vs. HD: *p* = 0.045). For the other PBUTs, the percentage change in plasma concentrations was not different between the HD- and HDF-treatment arms, and stratification according to RKF did not change this. Notably, residual renal creatinine clearance was stable during follow-up in the HD and HDF treatment arms. No association was observed between convection volume in HDF and the percentage change in PBUT plasma concentrations over time compared with HD (without convection) (Table 4).

### 2.4. PBUTs and All-Cause Mortality and Cardiovascular Event

After a median follow-up of 4.3 (2.3 to 5.9) years, 38 patients had died (48%) and a fatal or non-fatal cardiovascular event had occurred in 32 patients (40%).

Baseline PBUT plasma concentrations were not associated with all-cause mortality or cardiovascular events when analyzed as continuous variables (Table 5) or as PBUT tertiles (Table S4). No consistent association between either PBUT plasma concentrations at six months follow up (Table S5 and S6) or the percentage change in PBUT plasma concentration from baseline to six months follow up (Table S7) and risk of all-cause mortality and cardiovascular events was observed.

## 3. Discussion

In the present study, higher residual renal creatinine clearance was associated with lower plasma concentrations of kynurenic acid, indoxyl sulfate, indole-3-acetic acid, p-cresyl glucuronide and hippuric acid. Treatment with HDF only decreased indoxyl sulfate plasma concentrations as compared with low-flux HD after six months, and no association was observed between convection volume and the percentage change in PBUT plasma concentrations over time. No association was found between PBUT plasma concentrations and risk of all-cause mortality and new cardiovascular events.

Plasma concentrations of the PBUTs measured in our study population were higher than in the healthy population as reported in the literature, especially for the strongly protein bound toxins (Table 6) (kynurenine: 2.0-fold [23,24,25], kynurenic acid: 75-fold [26,27], indoxyl sulfate: 72-fold [28,29], indole-3-acetic acid: 2.4-fold [29], p-cresyl sulfate: 59-fold [29], p-cresyl glucuronide: 1.6-fold [30], and hippuric acid: 11-fold higher) due to impaired tubular secretion and limited removal during HD, since only the free fraction is available for diffusion across the dialysis membrane. Kynurenic acid, indoxyl sulfate, indole-3-acetic acid, p-cresyl glucuronide and hippuric acid concentrations were inversely related to renal creatinine clearance. Similarly, previous studies reported an inverse relation between RKF and indoxyl sulfate and p-cresyl sulfate plasma concentrations in end stage kidney disease (ESKD) patients [31,32,33,34,35]. The absence of a relation between kynurenine and p-cresyl sulfate concentrations and renal creatinine clearance in the present study may be explained by their relatively low affinity for the OAT receptor compared with other PBUTs [36], resulting in less OAT-mediated tubular secretion (Table 6). For p-cresyl glucuronide, which may also have lower affinity for OAT-1 [36] and OAT-3, residual glomerular filtration may have played a role, considering the high non-protein bound fraction in ESKD [37]. In addition, a high affinity of p-cresyl glucuronide for the efflux transporters MRP and BCRP located at the apical membrane of the proximal tubular cell, may theoretically explain the inverse relation with RKF [38]. By continuously transporting p-cresyl glucuronide out of the cell into the tubule lumen, a concentration gradient across the cell membrane is maintained, stimulating OAT-mediated transport of p-cresyl glucuronide into the tubule lumen, even when OAT-affinity of the substrate is low. Our findings are in line with Snauwaert et al. who found that loss of eGFR and residual urine volume was associated with an increase of indoxyl sulfate, hippuric acid, p-cresyl glucuronide, and indole-3-acetic acid plasma concentrations, and that the strength of the association increased with increasing affinity for the OAT receptor [39,40]. In agreement with our study, they found no association between p-cresyl sulfate and either eGFR or residual urine volume.

**Figure 2:**
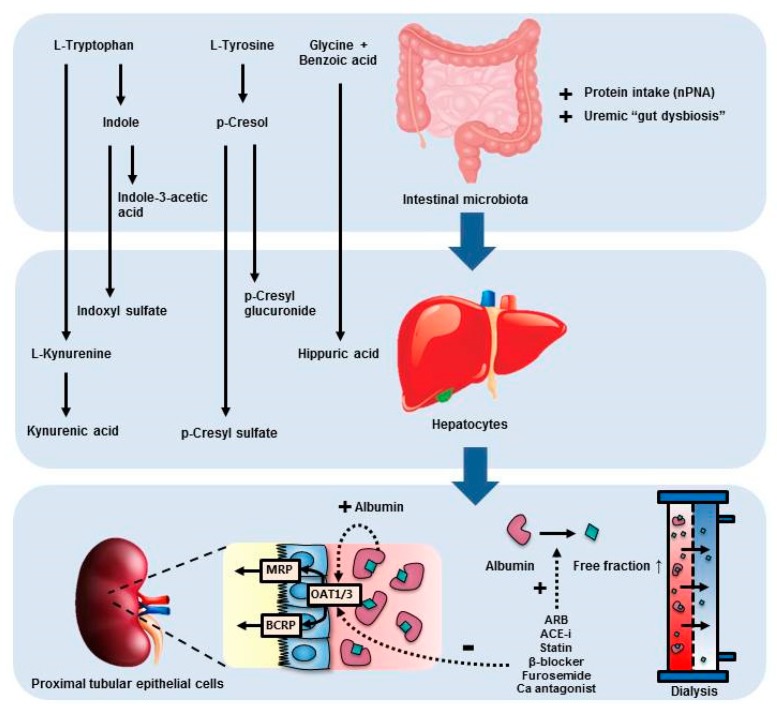
Metabolism and excretion of protein bound uremic toxins (PBUTs). The dietary amino acids L-tryptophan, L-tyrosine and glycine are metabolized by intestinal microbiotia and/or hepatocytes to form L-kynurenine, kynurenic acid, indoxyl sulfate, indole-3-acetic acid, p-cresyl sulfate, p-cresyl glucuronide and hippuric acid. In patients with chronic kidney disease, alterations in the composition and function of the intestinal microbiotia, so called “gut dysbiosis”, results in excess production of PBUTs. In the kidney, PBUTs are excreted via active tubular secretion by proximal tubular epithelial cells. Excretion is mediated by organic anion transporters (OAT) 1 and 3 that shift the albumin-PBUT binding toward the free fraction, allowing for active secretion of PBUTs in concerted action with MRP4 and BCRP [38]. Albumin facilitates active transport of PBUTs via the OAT1 transporter [36,45]. Removal of PBUTs during dialysis is limited because of their strong binding to plasma albumin, while only the free fraction can diffuse across the dialysis membrane. Medications may enhance dialytic removal of PBUTs by competitive binding to albumin, increasing the free PBUT fraction and its removal by dialysis. In the kidney however, medications may inhibit OAT-1 and -3 mediated transport, limiting renal PBUT secretion.

Several associations between PBUT plasma concentrations and patient characteristics were observed that can be related to PBUT metabolisms and their biological effects (Figure 2). We found an association between nPNA, a measure of dietary protein intake, and kynurenic acid and indoxyl sulfate. This may be explained by increased intestinal production of these solutes with higher intake of tryptophan. Dietary amino acids, such as tyrosine, tryptophan and glycine, undergo a series of reactions by bacterial fermentation, after which they are absorbed and transported via the portal circulation to the liver, where metabolization may occur (e.g., L-tryptophan is metabolized into L-kynurenine by tryptophan 2,3 dioxygenase and indole is metabolized to indoxyl sulfate by CYP-2E1 and sulfotransferase). The absence of a direct relation between dietary protein intake and kynurenine, hippuric acid, p-cresyl sulfate, p-cresyl glucuronide and indole-3-acetic acid, may reflect the particular composition of the microbiome and liver metabolism of our study population, that may both favor the production of kynurenic acid and indoxyl sulfate.

The role of albumin is complex as it is considered to hamper PBUT clearance by HD, but may facilitate active tubular secretion in the kidney. In the circulation, higher plasma albumin may result in more protein binding, limiting the availability of the free fraction for removal by dialysis. This may account for the direct relationship in patients without RKF between plasma albumin and p-cresyl sulfate, the PBUT with the highest protein bound fraction. In the kidney, however, higher plasma albumin may stimulate OAT-1-mediated transport of organic anions [36,45], which may result in lower PBUT concentrations in patients with RKF. These opposite effects of albumin on PBUT concentrations in patients with RKF may account for the absence of a consistent clinically relevant relationship between plasma albumin and PBUTs.

Medications may also have an opposite effect on dialytic and renal PBUT clearance by competitive binding to albumin and/or OAT inhibition, respectively. During HD, dissociation of bound PBUTs may be enhanced by compounds that competitively bind to the Sudlow I and II binding sites on human albumin, enhancing dialytic removal of the free fraction as was shown for furosemide in vitro after infusion into the bloodline upstream of the dialyzer [46]. Since plasma protein binding is high for ACE-inhibitors and most statins, these drugs may also cause PBUT displacement [47,48] and enhance dialytic removal. Thus, competitive binding to albumin may explain the inverse associations between furosemide use and kynurenic acid, ACE-inhibitor use and kynurenic acid and indole-3-acetic acid, and statin use and indole-3-acetic acid. On the other hand, medications, including statins, ARB and furosemide, may inhibit OAT-1 and -3 mediated tubular excretion by competitive transport, as has been demonstrated for indoxyl sulfate in vitro [49], and therefore increase PBUT plasma concentrations in patients with RKF. Accordingly, ARB use related directly to kynurenic acid concentrations in the present study.

Only predialysis indoxyl sulfate plasma concentrations decreased in patients treated with HDF compared with HD after six months of treatment, whereas no difference was observed for the other PBUTs measured in this study. In addition, no association was observed between convection volumes and change in PBUT plasma concentrations over time. Similarly, most studies applying HDF with convection volumes of 19-26 L and a follow up duration ranging from 1 week to 12 months did not find a decrease in predialysis concentrations of hippuric acid, indole-3-acetic acid, p-cresyl sulfate, p-cresyl glucuronide, or indoxyl sulfate compared with HD [18,19,21,50]. In contrast, Panichi et al. found a ~25%- and ~21%-decrease in indoxyl sulfate and p-cresyl sulfate plasma concentrations, respectively, after six months of treatment with postdilution HDF 24 L [22], and Meert et al. found a 20%-decrease in p-cresyl sulfate plasma concentrations after 9 weeks of treatment with postdilution HDF 19 L [17]. The discrepant findings between these studies might be explained by differences in dietary protein intake, composition and function of the gut microbiome, hepatic metabolism, medications, and renal clearance, which, besides dialytic clearance, are all important determinants of PBUT plasma concentrations, as illustrated in Figure 2. In support, Meijers et al. found that indoxyl sulfate and p-cresyl sulfate total serum concentrations were not related despite a strong correlation between dialytic clearances, suggesting that in addition to removal by hemodialysis, different metabolic pathways determine total serum concentrations [51]. Of note, as in the present study, all cited studies used HPLC or LC-MS for measurement of PBUT plasma concentrations.

Several factors may account for the absence of a reduction in predialysis PBUT plasma concentrations in the present study. First, the extent to which HDF may increase PBUT removal may be limited. In theory, HDF may improve PBUT clearance compared with low-flux HD via two mechanisms: (1) enhanced removal of the free fraction [52] and (2) enhanced removal of the bound-fraction via higher protein losses [53]. However, PBUTs in general have low molecular weights (<500 dalton, Table 6) and the extent to which HDF increases small solute clearance, including the free PBUT fraction, is limited because diffusive removal during HD is very efficient [54]. Furthermore, dissociation of bound PBUTs from plasma protein is too slow to restore equilibrium between the free and bound fractions during a dialysis session, especially for strongly bound PBUTs [42]. With regard to loss of the bound fraction, the effect of HDF may be limited. Although the filtered load of low molecular weight proteins is much higher with HDF than with low-flux HD [55], loss of albumin, which is the dominant plasma binding protein for anionic PBUTs [56], is only slightly higher [53] and does not correlate with PBUT concentrations in spent dialysate [18,52]. Meert et al. calculated that removal of bound PBUTs was <2% of total PBUT removal [52,57]. Thus, higher loss of albumin-bound PBUTs cannot account for enhanced PBUT removal with HDF. Second, the presence of continuous residual renal clearance in the majority of patients might have blunted the effect of improved dialytic PBUT clearance with HDF compared with HD on PBUT plasma concentrations in the total cohort. Even at very low values, renal clearance is a more important determinant of PBUT plasma concentrations than intermittent dialytic clearance, because of its continuous character [58,59]. Still, when only patients without RKF were analyzed, no effect of HDF on PBUT plasma concentrations could be detected, albeit that sample size was limited. Third, the delivered convection volume of 17.3 ± 4.3 L might be too low to increase removal of the free fraction to an extent that lowers PBUT plasma concentrations. Accordingly, Bammens et al. observed a 19%-decrease in p-cresyl sulfate and p-cresyl glucuronide predialyis plasma concentrations with predilution HDF 60 L compared with HD after two weeks of treatment, whereas no change was observed with postdilution HDF 20 L [18]. However, even when we stratified the change in PBUT plasma concentrations for tertiles of convection volume, we failed to observe a trend towards a decrease in PBUT plasma concentrations at higher convection volumes.

PBUT plasma concentrations at baseline were not associated with all-cause mortality and cardiovascular events [60]. In contrast, a meta-analysis of several small prospective studies indicated that elevated levels of free indoxyl sulfate and p-cresyl sulfate did associate with increased mortality in dialysis patients, while only p-cresyl sulfate was associated with an increased risk of cardiovascular events [12]. Furthermore, indoxyl sulfate and p-cresyl sulfate have been shown to play a role in the pathogenesis of cardiovascular disease in vitro and in vivo by inducing endothelial dysfunction, inflammation and oxidative stress [10,11]. There are several possible explanations for the absence of an association with outcome in the present study. First, the short dialysis vintage and high number of patients with RKF in the present study may have beneficially influenced outcome. Second, we measured total toxin concentrations, while free concentrations represent the biologically active fraction that can be removed by dialysis [61,62]. Indeed, Liabeuf et al. found that only free p-cresyl sulfate, but not the total concentration, was associated with mortality in patients with CKD [32]. Of note, Meijers et al. found that total and free serum concentrations were strongly correlated for either indoxyl sulfate and p-cresyl sulfate individually [51]. Third, dietary protein intake and PBUT plasma concentrations are directly correlated, but may have an opposite effect on outcome. In CKD patients, protein restriction has been reported to delay the progression of kidney disease [63,64,65], possibly mediated by reduced PBUT concentrations [32,66,67,68]. After initiation of HD, however, reduced protein intake is associated with higher all-cause mortality [69,70], which might be due to the inability to compensate for dialysis related protein loss and enhanced protein catabolism or because a low protein intake is characteristic of malnourished patients with a high comorbid burden. Adverse clinical outcome related to reduced protein intake may have masked the beneficial effects of lower PBUT plasma concentrations on outcome. Yet, adjustment for protein intake (nPNA) did not change the results. Last, the limited sample size may have precluded detection of significant associations between PBUT concentrations and mortality and cardiovascular events, especially in subgroup analysis.

One of the strengths of this study is that, to the best of our knowledge, this is the largest cohort of HDF patients in which PBUTs have been quantified with relatively long follow-up time. In addition, patients were well characterized, the study was randomized and seven different PBUTs were measured. The association between a variety of putative PBUT determinants, including medications, with PBUT plasma concentrations was evaluated. Of note, no mathematical correction was made for multiple comparison, but conclusions were only drawn based on significant associations that were consistent and/or observed for multiple PBUTs.

This study has several limitations. First, sample size was limited, which may account for the absence of an association between PBUTs and treatment modality and outcome. Second, dietary protein intake and drug prescription were not standardized and intestinal PBUT production was not quantified, both of which may have influenced PBUT plasma concentrations. Third, we measured total PBUT plasma concentrations while the free fraction represents the biologically active fraction that can be removed by dialysis. Last, PBUT concentrations were not measured in plasma after dialysis or in total collected spent dialysate, precluding calculation of PBUT reduction ratios and intradialytic PBUT removal and hence comparison of these parameters between HD and HDF.

## 4. Conclusions

In conclusion, residual kidney function is an important determinant of PBUT plasma concentrations in our population of HD patients. In our study, treatment with HDF for 6 months did not consistently decrease total PBUT plasma concentrations compared with HD. Despite the lack of a relation between measured PBUTs and mortality and cardiovascular events, we cannot exclude the fact that free PBUT plasma concentrations are associated with the outcome, or that accumulation of other PBUTs can increase the risk of (cardiovascular) endpoints. The current study suggests that alternative therapeutic strategies will be required to lower these PBUT plasma concentrations. Moreover, the complex relation of the individual PBUTs with baseline parameters stresses the importance of a therapeutic approach that focuses on intake, metabolism, distribution, and excretion when one aims to lower a specific, potentially pathogenic, PBUT.

## 5. Methods

### 5.1. Study Design and Patients

Data from the first 80 patients, enrolled between June 2004 and December 2009 in the CONvective TRAnsport STudy (CONTRAST), and in whom plasma samples at baseline and after 6 months of follow-up were available, were used. CONTRAST is a randomized multicenter-controlled trial (ISRCTN38365125) that compares the effects of low-flux HD versus online HDF on all-cause mortality and cardiovascular morbidity and mortality, as described [71]. The study was approved by the Medical Research Ethics Committee, VU Medical Center, Amsterdam, the Netherlands, on 3 December 2003 (ClinicalTrials gov identifier: NCT00205556). Patients were eligible for inclusion if they were treated with low-flux HD two or three times per week for at least 2 months, and were stable with a minimum dialysis single pool Kt/V urea (spKt/V_urea_) of 1.2. Exclusion criteria were being aged below 18 years, treatment with HDF or high-flux HD in the 6 months preceding randomization, a life expectancy of less than 3 months due to non-renal disease, participation in another clinical intervention trial evaluating a cardiovascular outcome, and severe non-adherence to the dialysis prescription (frequency and/or duration of dialysis treatment). Patients were randomized centrally by a computer-based randomization service (Julius Center University Medical Center, Utrecht, the Netherlands) into a 1:1 ratio and stratified per participating center (permuted blocks). The study was conducted in accordance with the Declaration of Helsinki and approved by the medical ethics review boards of all participating centers. Written informed consent was obtained from all patients prior to randomization.

### 5.2. Dialysis Procedures

Treatment times were fixed during follow-up in both treatment arms, unless spKt/V_urea_ was <1.2. Online HDF was performed in the post-dilution mode with a target convection volume of 6 L/h. Blood flow rates could be increased in the HDF arm to improve convection volumes. Synthetic high-flux dialyzers were used for HDF (FX80: 49% and FX100: 29% [Fresenius Medical Care, Bad Homburg, Germany], Polyflux 170H: 7% and Polyflux 210H: 10% [Gambro Corporation AB, Lund, Sweden], or other dialyzers: 5%, at 6 months). HD patients were treated with synthetic low-flux dialyzers (F8HPS: 71% and F10HPS: 5% [Fresenius], Polyflux 17 L: 17% and GFS Plos 20: 5% [Gambro]), except for one patient who used a high-flux dialyzer (FX80: 2% [Fresenius]). When HDF could temporarily not be performed due to technical reasons, the patients were treated with high-flux membranes. For both HDF and HD, ultrapure dialysis fluids were used, defined as less than 0.1 colony-forming units per mL and less than 0.03 endotoxin units per mL [72]. Routine patient care was performed according to national and international quality of care guidelines.

### 5.3. Data Collection

At baseline, demographical and clinical data were collected, including age, gender, body mass index (BMI, kg/m^2^), predialysis systolic and diastolic blood pressure (mmHg), predialysis pulse pressure (mmHg), medical history (including the cause of kidney disease, previous CVD and presence of diabetes mellitus (DM)), and medication use (beta blocker, calcium antagonist, angiotensin converting enzyme (ACE) inhibitor, angiotensin II receptor blocker (ARB), statin, and furosemide). Furthermore, dialysis related data were collected as described [71], including duration of dialysis (dialysis vintage), number of treatments per week, dialysis session duration, blood flow rate (mL/min), and normalized protein nitrogen appearance (nPNA, g/kg/d). History of CVD was defined as a history of angina pectoris, acute myocardial infarction, percutaneous transluminal coronary angioplasty, coronary artery bypass surgery, stroke, transient ischemic attack, intermittent claudication, amputation, percutaneous transluminal angioplasty, and peripheral bypass surgery. Dialysis vintage was determined as the sum of time spent on HD or peritoneal dialysis before inclusion in CONTRAST.

Residual kidney function was defined as a 24 h urine production >100 mL. In patients with a urinary production of >100 mL per day, interdialytic 24 h urinary samples were collected. 24 h renal creatinine clearance was used instead of eGFR, since creatinine clearance is dependent on both glomerular filtration active tubular secretion [73], and is thus also determined by residual tubular function which is important for PBUT secretion. Creatinine clearance of 24 h also showed a stronger association with PBUT concentrations than eGFR, calculated as the mean of urea and creatinine clearance. The plasma concentrations used for calculation of creatinine clearance were the mean of the values before and after dialysis. Renal creatinine clearance was considered zero in patients with a urinary production <100 mL per day. The second-generation Daugirdas formula was used to calculate spKt/V_urea_ [74].

Every 3 months during follow-up, data were recorded on clinical events, dialysis treatment, medication, and laboratory values. In HDF patients, infusion volumes (L per treatment) were reported as the mean value of three consecutive treatment sessions preceding the quarterly visit. Convection volumes (L per treatment) were calculated as the sum of the ultrafiltration volume and the substitution volume per session.

Blood samples for PBUT measurement were taken before the dialysis session at baseline and 6 months, immediately placed on ice, and centrifuged within 30 min, at 1500 relative centrifugal force for 10 min and stored at –80 °C until assayed.

### 5.4. Laboratory Analyses

Predialysis plasma concentrations of creatinine, phosphate, albumin, and hemoglobin were analyzed in the local laboratories of the participating hospitals. Creatinine, phosphate, and albumin in heparin plasma and creatinine in urine were analyzed on a DxC 800 routine chemistry system (Beckman Coulter, Brea, CA, USA). Creatinine was measured by a modified Jaffe-method, phosphate by an assay that is based on the reaction of phosphate with ammonium molybdate in an acidic solution to form a colored phosphomolybdate complex, and albumin was measured using the broom cresol green method. Hemoglobin was analyzed in EDTA blood on the CD-Sapphire routine hematology analyzer (Abbott Diagnostics, Santa Clara, CA, USA). High-sensitivity C reactive protein (CRP) was measured centrally with a particle-enhanced immunoturbidimetric assay on a Roche-Hitachi analyzer (Roche Diagnostics GmbH, Mannheim, Germany), with a lower quantification limit of 0.1 mg/L.

To measure predialysis total plasma concentrations of kynurenine (µmol/L), kynurenic acid (µmol/L), indoxyl sulfate (µmol/L), indole-3-acetic acid (µmol/L), p-cresyl sulfate (µmol/L), para-cresyl glucuronide (µmol/L), and hippuric acid (µmol/L), plasma samples were diluted and deproteinized by acetonitrile and analyzed centrally using ultrahigh performance liquid chromatography-mass spectroscopy (UHPLC-MS) analysis, modified from Poesen et al. [75] (see Supplementary Materials and Table S1). PBUT concentrations were quantified based on the peak area ratio of the sample and internal standard. In case of concentrations at the lower end of the quantifiable concentration range, weighted regression of 1/y^2^ was applied to obtain a more accurate concentration. Interpolation of unknowns from a calibration curve was performed using GraphPad Prism (version 6 for Windows, GraphPad Software, La Jolla California, CA, USA). If PBUT plasma concentrations exceeded the upper limit of quantification (ULOQ), the ULOQ was used for analysis (kynurenic acid: 5.0 µmol/L, hippuric acid: 1000 µmol/L, p-cresyl sulfate: 700 µmol/L, and p-cresyl glucuronide: 100 µmol/L).

### 5.5. Statistical Analysis

#### 5.5.1. Baseline Characteristics

Continuous variables are expressed as mean ± standard deviation (SD) when normally distributed, or as the median (interquartile range (IQR)) when non-normally distributed. Categorical variables are expressed as numbers (percentage). Unpaired student’s t-test was used for continuous variables with a normal distribution, Mann-Whitney U test for non-normally distributed continuous variables, and the Pearson Chi-Square test for categorical variables to compare patient characteristics at baseline between the HD and HDF group.

#### 5.5.2. Determinants of PBUT Plasma Concentrations at Baseline

To study the association between various patient characteristics, including RKF, and PBUT plasma concentrations at baseline, a backward multivariable regression model was applied with adjustment for age, gender, and 24 h renal creatinine clearance. Variables that might play a role in the production, metabolism, excretion, or pathogenesis of PBUT were selected. Adjustment for age and gender was applied because of their established relationship with cardiovascular risk. Adjustment for 24 h renal creatinine clearance was applied because a relationship with PBUT plasma concentrations was expected based on pathophysiological considerations. Subsequently, additional adjustment for dietary protein intake (nPNA) was applied since increased intestinal production of PBUTs with a higher intake of proteins may influence PBUT plasma concentrations.

The natural logarithm of hippuric acid, p-cresyl glucuronide, and indole-3-acetic acid was used to obtain a normal distribution that allowed parametric analysis. Results are expressed as beta (β) regression coefficients with *p*-value. The plasma concentration of kynurenine, kynurenic acid, and indoxyl sulfate would increase with β, and the plasma concentration of indole-3-acetic acid, p-cresyl glucuronide, and hippuric acid would be multiplied by exponent β (e^β^) if the variable increases by one unit or if the variable changes from 0 to 1 for a dichotomous variable. Because p-cresyl sulfate plasma concentrations exceeded the upper limit of detection in 28 cases, ordinal logistic regression analysis with p-cresyl sulfate tertiles was applied. Data are expressed as e^β^ with *p* values, which denotes the odds of a plasma concentration in a higher tertile if the variable increases by one unit or if the variable changes from 0 to 1 for a dichotomous variable.

The association between baseline PBUT concentrations and RKF was further explored by stratifying PBUT concentrations according to renal creatinine clearance (0 mL/min (n = 39), 0.1–2.4 mL/min (n = 14), 2.5–6.4 mL/min (n = 14), and >6.5 mL/min (n = 14)). One-way ANOVA with post hoc correction using Tukey’s honestly significant difference test was used for comparison of PBUT concentrations between strata. Because of the non-normal distribution, the natural logarithm of hippuric acid, p-cresyl sulfate, p-cresyl glucuronide, and indole-3-acetic acid was used.

The association between baseline PBUT plasma concentrations and plasma albumin and medications was further explored after stratification for RKF, since in patients with RKF albumin and medications may influence tubular secretion of PBUTs.

#### 5.5.3. PBUT Change over Time, HDF versus HD

A paired Student’s t-test or Wilcoxon signed rank test for non-normally distributed PBUTs was used to compare PBUT plasma concentrations between baseline and at the 6-month follow-up in the HD and HDF group.

The percentage change in PBUT plasma concentrations from baseline to follow-up was calculated as follows:

(1) $$\left(\%change/6months\right)=\frac{\left[Cp\right]t6-\left[Cp\right]t0}{\left[Cp\right]t0}\times 100\%,$$

where ∆ is the percentage change in PBUT plasma concentrations from baseline to 6 months (% change/6 months), [Cp]t0 is the toxin concentration at baseline, and [Cp]t6 is the toxin concentration at 6 months.

A Mann-Whitney U test was used to compare the percentage change (% change/6 months) in PBUT plasma concentrations between HDF and HD patients after 6 months of treatment. Finally, the relation between the percentage change in PBUT plasma concentrations and tertiles of the actual (on treatment) delivered convection volume was explored using one-way ANOVA.

A *P* value < 0.05 was considered significant. All analyses were performed with SPSS (version 18; SPSS Inc. Headquarters, Chicago, IL, USA).

#### 5.5.4. PBUTs and All-Cause Mortality and Cardiovascular Events

A Cox proportional hazards regression model was computed to estimate hazard ratios (HR) with 95% CI for all-cause mortality and the occurrence of a new fatal and non-fatal cardiovascular event censored for kidney transplantation associated with PBUT plasma concentrations at baseline and the percentage change in PBUT plasma concentrations from baseline to the 6-month follow-up. PBUT plasma concentrations were analyzed as continuous variables and divided into tertiles, of kynurenine (<3.6, 3.6–4.9 and >4.9 µmol/L), kynurenic acid (<1.5, 1.5–2.5 and >2.5 µmol/L), indoxyl sulfate (<123, 123–180 and >180 µmol/L), indole-3-acetic acid (<5.9, 5.9–9.2 and >9.2 µmol/L), p-cresyl sulfate (<337, <337–701 and >701 µmol/L), p-cresyl glucuronide (<5.1, 5.1–20.5 and >20.5 µmol/L), and hippuric acid (<141, 141–273 and >273 µmol/L). Cardiovascular events were defined as death from cardiovascular causes, nonfatal myocardial infarction or stroke, therapeutic coronary procedure (coronary artery bypass graft, percutaneous transluminal coronary angioplasty and/or stenting), therapeutic carotid procedure (endarterectomy and/or stenting), vascular intervention (revascularization, percutaneous transluminal angioplasty and/or stenting), or amputation. Two models were used. In Model I the unadjusted association between PBUT plasma concentrations and all-cause mortality or cardiovascular events was studied. In Model 2, adjustments were made for age, gender, diabetes mellitus, history of CVD, renal creatinine clearance, and nPNA, which are potential confounders of the relation between PBUT concentrations and all-cause mortality and cardiovascular events. Data for patients were censored at the date of the cardiovascular event or death or date of the last visit if patients were still alive at the end of follow-up (31 December 2010).

## Figures and Tables

**Table 1:** Baseline characteristics of the study population (*n* = 80).

Characteristic	Low-Flux HD (*n* = 41)	Online HDF (*n* = 39)	P^†^
Age – years	63.4 ± 13.1	62.4 ± 15.4	0.744
Male sex – no. (%)	25 (61)	20 (51)	0.499
Systolic blood pressure (mmHg)*	146 ± 19	147 ± 20	0.681
Diastolic blood pressure (mmHg)*	75 ± 10	77 ± 12	0.576
Pulse pressure (mmHg)*	70 ± 17	70 ± 16	0.913
History of CVD – no. (%)	18 (44)	16 (41)	0.824
Diabetes mellitus – no. (%)	8 (20)	9 (23)	0.788
24-h urine volume (mL)	550 (305–1000)	798 (246–1295)	0.478
Residual kidney function – no. (%)^‡^	20 (49)	24 (62)	0.271
Renal creatinine clearance (mL/min)	0.0 (0.0–3.6)	1.5 (0.0–5.9)	0.249
Dialysis vintage (years)	2.3 (0.7–3.7)	1.4 (0.8–2.4)	0.182
Normalized protein equivalent of nitrogen appearance (g/kg/d)	1.19 ± 0.24	1.14 ± 0.24	0.409
Beta blocker – no. (%)	23 (56)	15 (39)	0.125
Calcium antagonist – no. (%)	15 (37)	12 (31)	0.641
ACE-inhibitor – no. (%)	17 (43)	12 (31)	0.352
ARB – no. (%)	8 (20)	13 (33)	0.210
Statin – no. (%)	19 (46)	25 (64)	0.123
Furosemide– no. (%)	7 (18)	14 (36)	0.078
Creatinin (µmol/L)*	965 ± 211	886 ± 264	0.143
Phosphate (mmol/L)*	1.76 ± 0.52	1.72 ± 0.49	0.711
Albumin (g/L)*	41.6 ± 3.9	41.6 ± 3.0	0.968
HsCRP (mg/L)*	10.5 ± 17.6	8.2 ± 16.2	0.561
Hemoglobin (mmol/L)*	7.3 ± 0.6	7.6 ± 0.8	0.183
Kynurenine (µmol/L)*	4.1 ± 1.2	4.4 ± 1.5	0.383
Kynurenic acid (µmol/L)*	2.4 ± 1.2	1.9 ± 1.1	0.023
Indoxyl sulfate (µmol/L)*	174 ± 59	143 ± 72	0.044
Indole-3-acetic acid (µmol/L)*	7.3 (5.1–15.0)	6.6 (5.3–12.9)	0.667
p-Cresyl sulfate*	618 (311–701)	527 (195–701)	0.449
p-Cresyl glucuronide (µmol/L)*	14.0 (5.1–32.1)	8.9 (2.5–23.9)	0.120
Hippuric acid (µmol/L)*	201 (103–333)	182 (95–295)	0.487

Continuous variables are expressed as mean ± standard deviation (SD) when normally distributed, median (interquartile range (IQR)) when non-normally distributed, and absolute numbers (%) for categorical data. ACE-inhibitor, angiotensin-converting enzyme inhibitor; ARB, angiotensin II receptor blocker; CVD, cardiovascular disease; hsCRP, high-sensitivity C-reactive protein. ^†^ Unpaired student’s t-test was used for continuous variables with a normal distribution, Mann-Whitney U test for non-normally distributed continuous variables, and Pearson Chi-Square test for categorical variables, significant differences are shown in bold font. * Predialysis; ^‡^ Residual kidney function was defined as a 24-h urine production >100 mL.

**Table 2:** Association between patient characteristics and protein-bound uremic toxin concentrations at baseline.

Characteristic	Kynurenine (µmol/L)*	Kynurenic Acid (µmol/L)*	Indoxyl Sulfate (µmol/L)*	Indole-3-Acetic Acid (µmol/L)*	p-Cresyl Sulfate tertiles^†^	p-Cresyl Glucuronide (µmol/L)*	Hippuric Acid (µmol/L)*
	β (*p*)	β (*p*)	β (*p*)	e^β^ (*p*)	e^β^ (*p*)	e^β^ (*p*)	e^β^ (*p*)
Age (years)	−0.003 (0.808)	**−0.020 (0.020)**	−0.167 (0.754)	1.000 (0.976)	1.010 (0.531)	1.001 (0.895)	1.001 (0.899)
Gender (male = 1)	0.203 (0.514)	0.356 (0.145)	3.750 (0.802)	1.180 (0.309)	1.784 (0.179)	0.668 (0.182)	1.062 (0.726)
Renal creatinine clearance (mL/min/1.73m^2^)	−0.019 (0.514)	**−0.115 (<0.001)**	**−6.024 (0.001)**	**0.958 (0.024)**	1.002 (0.968)	**0.899 (0.004)**	**0.898 (<0.001)**
nPNA (g/kg/d)	−1.579 (0.055)	**2.367 (<0.001)**	**110.055 (0.005)**	0.852 (0.687)	4.242 (0.216)	2.572 (0.236)	1.891 (0.148)
Dialysis vintage (years)	−0.096 (0.102)	−0.003 (0.951)	−0.311 (0.914)	0.950 (0.077)	1.122 (0.187)	1.082 (0.170)	0.984 (0.613)
Albumin (g/L)	0.027 (0.543)	0.054 (0.119)	0.019 (0.993)	1.047 (0.072)	**1.216 (0.004)**	1.082 (0.065)	1.019 (0.462)
Beta blocker (yes = 1)	0.334 (0.282)	0.418 (0.084)	19.877 (0.184)	0.976 (0.881)	1.507 (0.341)	0.851 (0.594)	1.189 (0.314)
Calcium antagonist (yes = 1)	0.068 (0.833)	0.018 (0.942)	−12.860 (0.407)	1.122 (0.500)	1.084 (0.855)	0.689 (0.231)	0.752 (0.113)
ACE-inhibitor (yes = 1)	−0.041 (0.901)	**−0.550 (0.028)**	13.994 (0.374)	0.724 (0.052)	1.443 (0.458)	1.235 (0.505)	0.800 (0.214)
ARB (yes = 1)	0.008 (0.983)	**0.653** **(0.018)**	−16.456 (0.344)	1.065 (0.727)	0.789 (0.595)	0.800 (0.525)	1.337 (0.141)
Statin (yes = 1)	0.039 (0.902)	0.224 (0.359)	3.831 (0.800)	**0.705 (0.030)**	0.908 (0.823)	1.061 (0.845)	0.800 (0.196)
Furosemide (yes = 1)	0.440 (0.210)	**−0.685 (0.011)**	−14.406 (0.399)	1.072 (0.714)	1.770 (0.242)	0.702 (0.301)	1.114 (0.580)
Systolic blood pressure (mmHg)	0.001 (0.857)	0.007 (0.278)	−0.230 (0.547)	0.993 (0.126)	0.988 (0.292)	1.001 (0.862)	1.001 (0.804)
Diastolic blood pressure (mmHg)	−0.004 (0.776)	0.010 (0.401)	0.790 (0.277)	0.994 (0.463)	1.016 (0.459)	1.015 (0.319)	1.013 (0.120)
Pulse pressure (mmHg)	0.004 (0.690)	0.006 (0.438)	−0.650 (0.156)	0.993 (0.165)	0.977 (0.088)	0.996 (0.676)	0.997 (0.504)
History of CVD (yes = 1)	0.248 (0.468)	0.069 (0.797)	17.557 (0.286)	0.863 (0.407)	1.842 (0.198)	0.847 (0.617)	0.724 (0.087)
Diabetes mellitus (yes = 1)	0.158 (0.693)	**0.624** **(0.044)**	27.092 (0.159)	1.047 (0.843)	1.322 (0.614)	0.704 (0.367)	1.492 (0.074)
HsCRP (mg/L)	−0.001 (0.911)	−0.010 (0.177)	0.585 (0.216)	0.999 (0.917)	0.968 (0.076)	0.994 (0.486)	0.999 (0.898)

ACE-inhibitor, angiotensin-converting enzyme inhibitor; ARB, angiotensin II receptor blocker; CVD, cardiovascular disease; HsCRP, high-sensitivity C-reactive protein; nPNA, normalized protein nitrogen appearance. * Multivariate linear regression analysis with adjustment for age, gender and renal creatinine clearance. Data are expressed as the unstandardized beta regression coefficient (β) with *p*-value, significant relations are shown in bold font. The plasma concentration of kynurenine, kynurenic acid and indoxyl sulfate will increase with β, and the plasma concentration of indole-3-acetic acid, p-cresyl glucuronide and hippuric acid, will be multiplied by e^β^ if the variable increases by 1 unit or if the variable changes from 0 to 1 for a dichotomous variable. **^†^** Ordinal logistic regression analysis with adjustment for age, gender and creatinine clearance for p-cresyl sulfate tertiles (tertile 1: <337 µmol/L, tertile 2: 337–701 µmol/L, tertile 3: >701 µmol/L). Data are expressed as e^β^ with *p*-value which denotes the odds of having a plasma concentration in a higher tertile if the variable increases by 1 unit or changes from 0 to 1 for a dichotomous variable. Significant associations are shown in bold font.

**Table 3:** Percentage change in protein-bound uremic plasma concentrations over time in patients treated with hemodialysis and hemodiafiltration stratified for patients with and without residual kidney function.

PBUT	RKF	*HD*			*HDF*			*HD* vs. *HDF*
		N	∆ (% Change/6 Months)	p*	N	∆ (% Change/6 Months)	p*	p**
Kynurenine	all	38	−7.7 (−22.6 to 14.5)	0.269	35	−5.9 (−20.9 to 29.3)	0.694	0.453
	NO	19	−9.4 (−21.8 to 12.4)	0.171	12	−19.6 (−42.2 to 5.6)	**0.019**	0.181
	YES	19	−5.9 (−30.1 to 25.7)	0.693	23	17.6 (−13.5 to 17.5)	0.226	0.146
Kynurenic acid	all	38	5.6 (−8.6 to 69.1)	0.111	36	3.2 (−22.1 to 39.5)	0.537	0.430
	NO	19	10.0 (−8.5 to 68.8)	0.414	12	−6.1 (−35.2 to 28.5)	0.729	0.256
	YES	19	1.6 (−9.1 to 70.1)	0.141	24	12.8 (−14.0 to 43.8)	0.150	0.883
Indoxyl sulfate	all	38	11.9 (−15.4 to 31.9)	0.133	36	−8.0 (−34.6 to 15.3)	0.092	**0.045**
	NO	19	14.5 (−14.3 to 31.7)	0.130	12	4.6 (−19.6 to 19.6)	0.992	0.351
	YES	19	1.7 (−21.3 to 33.9)	0.524	24	−17.8 (−48.0 to 10.9)	0.075	0.129
Indole-3-acetic acid	all	25	9.2 (−19.6 to 34.9)	0.876	27	−10.8 (−26.0 to 14.0)	0.615	0.356
	NO	12	0.6 (−36.3 to 25.9)	0.721	10	−15.1 (−28.3 to 5.3)	0.314	0.568
	YES	13	10.9 (−15.2 to 40.7)	0.477	17	5.8 (−25.6 to 52.5)	0.861	0.434
p-Cresyl sulfate	all	38	−8.8 (−28.9 to 29.5)	0.510	36	−2.7 (−27.4 to 10.2)	0.199	0.854
	NO	19	−7.3 (−24.2 to 17.6)	0.381	12	2.6 (−21.7 to 86.9)	0.859	0.394
	YES	19	−10.7 (−31.8 to 79.5)	0.906	24	−4.0 (−40.3 to 0.00)	0.053	0.477
p-Cresyl glucuronide	all	38	−7.0 (−38.1 to 69.8)	0.421	36	7.4 (−37.3 to 65.3)	0.765	0.681
	NO	19	−6.4 (−43.6 to 39.2)	0.077	12	30.5 (−33.5 to 181.0)	0.239	0.096
	YES	19	−15.5 (−36.6 to 154.6)	0.520	24	−2.8 (−53.5 to 50.0)	0.710	0.478
Hippuric acid	all	38	5.7 (−44.6 to 54.5)	0.531	36	−21.9 (−47.6 to 42.4)	0.187	0.566
	NO	19	11.1 (−42.6 to 59.9)	0.557	12	−11.1 (−47.5 to 75.3)	0.583	0.832
	YES	19	5.7 (−47.7 to 53.1)	0.778	24	−29.6 (−47.6 to 42.4)	0.199	0.696

Data are presented as median (interquartile range). * *P* values were calculated comparing plasma concentrations at baseline vs. 6 months using a Students paired t-test or Wilcoxon signed rank test as appropriate. ** *P* values were calculated using a Mann-Whitney U test, significant p-values are shown in bold font. HD, low-flux hemodialysis; HDF, hemodiafiltration; PBUT, protein-bound uremic toxin; RKF, residual kidney function; ∆, percentage change in PBUT plasma concentrations from baseline to 6-months of follow-up (% change/6 months).

**Table 4:** Effect of convection volume on the percentage change in plasma protein-bound uremic toxins over time.

PBUT	Convection Volume (L)	N	∆ (% Change/6 Months)	p*
Kynurenine	< 14.3	11	14.6 ± 42.6	0.265
	14.3–18.4	14	8.5 ± 38.7	
	>18.4	12	−9.8 ± 29.1	
Kynurenic acid	< 14.3	12	10.8 ± 42.9	0.972
	14.3–18.4	14	15.3 ± 42.6	
	>18.4	12	14.0 ± 46.7	
Indoxyl sulfate	< 14.3	12	6.8 ± 48.8	0.774
	14.3–18.4	14	−17.7 ± 37.3	
	>18.4	12	−3.9 ± 40.4	
Indole-3-acetic acid	< 14.3	7	21.2 ± 61.1	0.453
	14.3–18.4	9	−1.3 ± 35.9	
	>18.4	7	−7.4 ± 32.7	
P-cresyl sulfate	< 14.3	12	−2.1 ± 65.7	0.597
	14.3–18.4	14	1.8 ± 64.4	
	>18.4	12	54.0 ± 252.8	
P-cresyl glucuronide	< 14.3	12	41.8 ± 91.4	0.541
	14.3–18.4	14	38.5 ± 135.7	
	>18.4	12	233.7 ± 868.1	
Hippuric acid	< 14.3	12	23.5 ± 92.3	0.345
	14.3–18.4	14	−2.0 ± 89.2	
	>18.4	12	11.1 ± 89.8	

Data are expressed as mean ± standard deviation. ∆, percentage change in protein-bound uremic toxin (PBUT) plasma concentrations from baseline to 6-months of follow-up (% change/6 months). * One-way ANOVA.

**Table 5:** Hazard ratios for all-cause mortality and new cardiovascular events for PBUT plasma concentrations at baseline.

				Hazard Ratio (95% CI)			
PBUT	Outcome	N	# Events	Model I	*P*	Model II	*P*
Kynurenine (µmol/L)	All-cause mortality	79	34	1.020 (0.802 to 1.298)	0.872	0.943 (0.707 to 1.256)	0.687
	CV events	78	29	1.054 (0.807 to 1.376)	0.701	0.982 (0.717 to 1.346)	0.911
Kynurenic acid (µmol/L)	All-cause mortality	80	35	0.879 (0.638 to 1.210)	0.429	1.104 (0.666 to 1.829)	0.702
	CV events	79	29	0.876 (0.622 to 1.235)	0.876	1.333 (0.798 to 2.226)	0.272
Indoxyl sulfate (µmol/L)	All-cause mortality	80	35	1.001 (0.995 to 1.006)	0.837	1.002 (0.995 to 1.009)	0.617
	CV events	79	29	1.003 (0.998 to 1.008)	0.290	1.007 (1.000 to 1.015)	0.056
Indole-3-acetic acid (µmol/L)	All-cause mortality	60	24	1.190 (0.609 to 2.323)	0.610	1.346 (0.568 to 3.192)	0.500
	CV events	59	20	1.002 (0.493 to 2.039)	0.995	1.434 (0.535 to 3.847)	0.474
p-Cresyl sulfate (µmol/L)	All-cause mortality	80	35	0.955 (0.670 to 1.362)	0.801	0.897 (0.614 to 1.310)	0.574
	CV events	79	29	0.960 (0.664 to 1.389)	0.829	1.036 (0.667 to 1.611)	0.874
p-Cresyl glucuronide (µmol/L)	All-cause mortality	80	35	0.992 (0.767 to 1.283)	0.952	1.024 (0.782 to 1.340)	0.864
	CV events	79	29	1.032 (0.775 to 1.374)	0.830	1.189 (0.863 to 1.636)	0.289
Hippuric acid (µmol/L)	All-cause mortality	78	33	1.037 (0.688 to 1.562)	0.862	0.966 (0.569 to 1.642)	0.900
	CV events	77	28	0.722 (0.457 to 1.142)	0.164	0.823 (0.445 to 1.524)	0.536

Uni/Multivariate Cox proportional hazards regression analysis censored for kidney transplantation. Model I: univariate; model II: adjustment for age, gender, diabetes mellitus, history of cardiovascular disease, renal creatinine clearance and normalized protein nitrogen appearance. Data are expressed as hazard ratios with 95% confidence interval (CI) and *p*-value for the occurrence of new cardiovascular (CV) events and all-cause mortality associated with a one-unit increase in PBUT plasma concentration.

**Table 6:** Physicochemical characteristics of PBUTs.

	Kyn	KA	IS	IAA	pCG	pCS	HA
OAT affinity [1,36]*	-	++++	++	+	--	+/-	+++
Molecular weight (g/mol) [41]	208.21	189.17	213.21	175.18	284.26	188.2	179.17
Protein binding (%) [42,43,44]	67	95	87-98	53-69	12-13	95	39-41
Water solubility (mg/L) [41]	1.67	0.95	0.79	1.38	24	1.58	1.18
pKa [41]	1.2	3.2	−1.8	4.7	3.3	−2.0	3.6

HA, hippuric acid; IAA, indole-3-acetic acid; IS, indoxyl sulfate; KA, kynurenic acid; Kyn, kynurenine; OAT, organic anion transporter; pCG, p-cresyl glucuronide; pCS, p-cresyl sulfate; pKa, acid dissocation constant. * OAT affinity for protein-bound uremic toxins is graded from relatively weak to strong (--, -, +/-, +, ++, +++, ++++).

## Supplementary Materials

The following are available online at https://www.mdpi.com/2072-6651/12/4/234/s1, Table S1: LC-MS parameters for the quantification of PBUTs, Table S2: Association between patient characteristics and protein bound uremic toxin concentrations at baseline corrected for normalized protein nitrogen appearance (nPNA), Table S3: Association between medications and protein bound uremic toxin concentrations at baseline stratified for residual kidney function, Table S4: Hazard ratios for all-cause mortality and new cardiovascular events for PBUT tertiles at baseline, Table S5: Hazard ratios for all-cause mortality and new cardiovascular events for PBUT plasma concentrations at follow up, Table S6: Hazard ratios for all-cause mortality and new cardiovascular events for PBUT tertiles at follow up, Table S7: Hazard ratios for all-cause mortality and new cardiovascular events for the percentage change in PBUT plasma concentrations from baseline to follow up.
