# Supplementary material for: Protein-Bound Uremic Toxins in Hemodialysis Patients Relate to Residual Kidney Function, Are Not Influenced by Convective Transport, and Do Not Relate to Outcome
Source: Toxins (Basel). 2020 Apr 7;12(4):234. doi: 10.3390/toxins12040234 (PMC7232478; doi:10.3390/toxins12040234)
Supplement: Supplementary file 1 [file toxins-12-00234-s001.pdf]

Supplementary Materials: Protein-Bound Uremic Toxins in Hemodialysis Patients Relate to Residual Kidney Function, are Not Influenced by Convective Transport, and Do Not Relate to Outcome

Maaike K. van Gelder, Igor R. Middel, Robin W.M. Vernooij, Michiel L. Bots, Marianne C. Verhaar, Rosalinde Masereeuw, Muriel P. Grooteman, Menso J. Nubé, M. A. van den Dorpel, Peter J. Blankestijn, Maarten B. Rookmaaker and Karin G.F. Gerritsen

Concise Methods for PBUT Measurement

Chemicals and Reagents

Kynurine, kynurenic acid, indoxyl sulfate (IS), indole-3-acetic acid (IAA), hippuric acid (HA), acetic acid (HAc; LC-MS grade) and dimethyl sulfoxide (DMSO; 99.9%) were purchased from Sigma-Aldrich (St. Louis, MO, USA). p-Cresyl sulfate p-Cresyl glucuronide and were purchased from AlsaChim (Illkirch-Graffenstaden, France). Acetonitrile (ACN; LC-MS grade) was obtained from Biosolve BV (Valkenswaard, the Netherlands). The isotopically labeled internal standards d_4-kynurenine, d_5-kynurenic acid and d_5-indole-3-acetic acid were obtained from CDN Isotopes (Pointe-Claire, Quebec, Canada), $13C_6$-indoxyl sulfate and d_5-hippuric acid and were purchased from Cambridge Isotope Laboratories (Andover, MA, USA), d_7-p-cresyl sulfate (potassium salt) was purchased from IsoSciences (Ambler, PA, USA) and d_7-p-cresyl glucuronide was purchased from Toronto Research Chemicals (North York, Ontario, Canada). Ultra-pure water (Synergy® Water Purification System) was used to prepare all aqueous solutions, dilutions, and eluents.

Sample Work-Up

Plasma samples were processed prior to LC-MS analysis. A 100-µL sample (plasma or surrogate matrix) was pipetted into a 1-mL 96-well plate. Phosphate-buffered saline (pH 7.4) was used as a surrogate matrix for calibration curve and quality control samples. Plasma samples were diluted by addition of 50 µL of water. For calibration curve and quality control samples, the 50-µL water sample contained the standards. Subsequently, 400 µL of cold (4 °C) ACN with internal standards d_4-kynurenine (1 µmol/L), d_5-kynurenic acid (0.5 µmol/L), $13C_6$-indoxyl sulfate (2 µmol/L), d_5- indole-3-acetic acid (2 µmol/L), d_7-p-cresyl sulfate (2 µmol/L), d_7-p-cresyl glucuronide (2 µmol/L) and d_5-hippuric acid (2 µmol/L) was added. Samples were vortexed for 5 minutes on a plate vortexer and centrifuged for 5 minutes 2643 RCF. A 400-µL sample of the supernatant was transferred to a new plate and dried under a nitrogen flow at 40°C. The residue was reconstituted in 280 µL of water and centrifuged again for 5 minutes at 2643 RCF. An 80-µL sample was transferred into a 96-well plate of which 7.5 µL was injected for UHPLC-MS analysis.

Liquid Chromatography-Mass Spectrometry

A 1290 Infinity UHPLC system (Agilent Technologies, Waldbronn, BW, Germany) coupled to an Agilent 6560 ion mobility quadrupole time-of-flight mass spectrometer (IM-QToF) was used. A Waters ACQUITY UPLC HSS T3 column (100 mm × 2.1 mm, 1.8 µm particles) combined with an ACQUITY UPLC HSS T3 VanGuard pre-column (5 mm × 2.1 mm, 1.8 µm particles) was used and was kept at a temperature of 40 °C. Mixed isocratic and gradient elution at a flow rate of 0.35 mL/min was applied for analyte separation. In the mobile phase, solvent A consisted of 10 mmol/L HAc, and solvent B was ACN. The final method was an initial isocratic composition of 5% B held for 1 min, followed by a linear increase

to 15% B in 1 min, and to 20% B in another 1 min. Then, B was increased linearly to 100% over 2 min, and was held at 100% for 0.5 min to flush the column. To re-equilibrate, B was reduced back to the initial isocratic composition of 5% over 1.5 min and was held for 4 min. The MS settings were ionization, capillary and nozzle voltage, nebulizing and drying gas flow and temperature, and the tuned mass range (Table S1).

Table S1. LC-MS parameters for the quantification of PBUTs.

PBUT / Internal Standard	Parent Ion (m/z)	Fragment Ion (m/z)	Retention Time (min)	LLOQ (µmol/L)	ULOQ (µmol/L)
Kynurenine	207.08	**144.04**	2.1	1.0	20
d_4-kynurenine	211.12	**148.07**	2.1		
Kynurenic acid	188.03	**144.04**	3.2	0.10	5.0
d_5-kynurenic acid	193.06	**149.08**	3.2		
Indoxyl sulfate	**212.00**		4.5	0.4	400
$13C_6$-indoxyl sulfate	**218.02**		4.5		
Indole-3-acetic acid	174.05	**144.04**	5.8	0.5	50
d_5- indole-3-acetic acid	179.09	**149.08**	5.8		
P-cresyl sulfate	**187.00**		5.8	4	700
d_7-p-cresyl sulfate	**194.05**		5.8		
P-cresyl glucuronide	**283.08**		4.6	0.1	100
d_7-p-cresyl glucuronide	**290.13**		4.6		
Hippuric acid	**178.05**		3.9	1.0	1000
d_5-hippuric acid	**183.08**		3.9		

All analytes were measured in negative mode. Kynurenine, kynurenic acid and indole-3-acetic acid ions underwent major MS-fragmentation. Bold, underlined masses indicate the ions used for quantification for the respective analyte. LLOQ, lower limit of quantification; ULOQ, upper limit of quantification.

Table S2. Association between patient characteristics and protein bound uremic toxin concentrations at baseline corrected for normalized protein nitrogen appearance.

	Kynurenine (µmol/L)*	Kynurenic Acid (µmol/L)*	Indoxyl Sulfate (µmol/L)*	Indole-3-Acetic Acid (µmol/L)*	p-Cresyl Sulfate Tertiles†	p-Cresyl Glucuronide (µmol/L)*	Hippuric Acid (µmol/L)*
**Characteristic**	**β (*p*)**	**β (*p*)**	**β (*p*)**	**$e^{β}$ (*p*)**	**$e^{β}$ (*p*)**	**$e^{β}$ (*p*)**	**$e^{β}$ (*p*)**
Age (years)	-0.006 (0.597)	-0.012 (0.132)	0.116 (0.831)	0.999 (0.822)	1.012 (0.476)	1.005 (0.635)	1.000 (0.989)
Gender (male = 1)	0.009 (0.979)	**0.445 (0.043)**	6.787 (0.658)	**1.473 (0.017)**	1.850 (0.196)	0.668 (0.210)	1.072 (0.696)
Renal creatinine	0.013 (0.800)	**-0.198 (<0.001)**	**-11.139 (<0.001)**	0.960 (0.106)	1.066 (0.400)	**0.903 (0.043)**	**0.843 (<0.001)**

clearance (mL/min/1.73m^2)							
nPNA (g/kg/d)	-1.579 (0.055)	**2.367 (<0.001)**	**110.005 (0.005)**	0.852 (0.687)	5.094 (0.169)	2.572 (0.236)	1.891 (0.148)
Dialysis vintage (years)	-0.101 (0.093)	0.009 (0.827)	0.130 (0.963)	**0.949 (0.049)**	1.113 (0.234)	1.072 (0.237)	1.000 (0.999)
Albumin (g/L)	0.030 (0.516)	0.040 (0.184)	0.227 (0.916)	**1.057 (0.024)**	**1.179 (0.018)**	1.065 (0.157)	1.026 (0.301)
Beta blocker (yes = 1)	0.501 (0.131)	0.238 (0.273)	8.099 (0.600)	0.924 (0.620)	1.322 (0.553)	0.888 (0.713)	1.086 (0.643)
Calcium antagonist (yes = 1)	0.155 (0.657)	-0.136 (0.553)	-20.643 (0.200)	1.168 (0.362)	0.940 (0.900)	0.687 (0.267)	0.754 (0.136)
ACE-inhibitor (yes = 1)	-0.329 (0.363)	-0.325 (0.159)	**31.824 (0.049)**	0.781 (0.136)	0.643 (0.379)	1.457 (0.274)	0.861 (0.434)
ARB (yes = 1)	0.167 (0.690)	0.435 (0.110)	-27.416 (0.155)	1.028 (0.885)	2.067 (0.228)	0.661 (0.309)	**1.657 (0.021)**
Statin (yes = 1)	0.172 (0.624)	0.137 (0.549)	3.524 (0.828)	0.807 (0.193)	1.080 (0.874)	0.952 (0.884)	0.825 (0.297)
Furosemide (yes = 1)	0.369 (0.313)	**-0.573 (0.014)**	-10.800 (0.524)	1.132 (0.490)	2.306 (0.109)	0.670 (0.259)	1.105 (0.609)
Systolic blood pressure (mmHg)	0.002 (0.786)	0.010 (0.078)	-0.326 (0.407)	0.998 (0.655)	0.992 (0.489)	1.004 (0.601)	1.001 (0.754)
Diastolic blood pressure (mmHg)	-0.002 (0.876)	0.014 (0.156)	0.822 (0.246)	0.996 (0.632)	1.018 (0.405)	1.017 (0.260)	1.013 (0.104)
Pulse pressure (mmHg)	0.005 (0.659)	0.008 (0.236)	-0.871 (0.068)	0.999 (0.832)	0.980 (0.173)	0.999 (0.904)	0.996 (0.483)
History of CVD (yes = 1)	0.180 (0.616)	0.030 (0.897)	16.841 (0.307)	1.005 (0.975)	2.291 (0.105)	0.754 (0.414)	0.700 (0.060)
Diabetes mellitus (yes = 1)	0.357 (0.412)	0.432 (0.127)	26.225 (0.191)	0.801 (0.347)	1.431 (0.563)	0.716 (0.429)	1.523 (0.072)
HsCRP (mg/L)	0.001 (0.916)	-0.011 (0.085)	3.396 (0.376)	1.000 (0.991)	0.973 (0.082)	0.995 (0.547)	0.997 (0.513)

ACE-inhibitor, angiotensin-converting enzyme inhibitor; ARB, angiotensin II receptor blocker; CVD, cardiovascular disease; HsCRP, high-sensitivity C-reactive protein; nPNA, normalized protein nitrogen appearance. *Multivariate linear regression analysis with adjustment for age, gender, renal creatinine clearance and normalized protein nitrogen appearance. Data are expressed as the unstandardized beta regression coefficient (β) with *p*-value. The plasma concentration of kynurenine, kynurenic acid and indoxyl sulfate will increase with β, and the plasma concentration of indole-3-acetic acid, p-cresyl glucuronide and hippuric acid, will be multiplied by e^{β} if the variable increases by 1 unit or if the variable changes from 0 to 1 for a dichotomous variable. †Ordinal logistic regression analysis with adjustment for age, gender and creatinine clearance for p-cresyl sulfate tertiles (tertile 1: <337 µmol/L, tertile 2: 337-701 µmol/L, tertile 3: >701 µmol/L). Data are expressed as e^{β} with *p*-value which denotes the odds of having a plasma concentration in a higher tertile if the variable increases by 1 unit or changes from 0 to 1 for a dichotomous variable. Significant associations are shown in bold font.

Table S3. Association between medications and protein bound uremic toxin concentrations at baseline stratified for residual kidney function.

Characteristic	Kynurenine (μmol/L)* β (*p*)	Kynurenic acid (μmol/L)* β (*p*)	Indoxyl sulfate (μmol/L)* β (*p*)	Indole-3-acetic acid (μmol/L)* e^{β} (*p*)	p-Cresyl sulfate tertiles† e^{β} (*p*)	p-Cresyl glucuronide (μmol/L)* e^{β} (*p*)	Hippuric acid (μmol/L)* e^{β} (*p*)
**Patients without RKF (*n* = 36)**							
Albumin (g/L)	0.032 (0.059)	0.048 (0.306)	-1.428 (0.637)	1.049 (0.208)	**1.275 (0.010)**	0.096 (0.387)	1.021 (0.503)
Beta blocker (yes = 1)	0.369 (0.373)	0.227 (0.557)	0.152 (0.050)	0.712 (0.190)	1.714 (0.396)	1.271 (0.623)	1.043 (0.863)
Calcium antagonist (yes = 1)	0.106 (0.810)	-0.137 (0.738)	2.347 (0.928)	0.896 (0.702)	0.617 (0.468)	1.096 (0.859)	0.755 (0.330)
ACE-inhibitor (yes = 1)	-0.191 (0.692)	-0.612 (0.165)	24.820 (0.383)	0.652 (0.125)	0.794 (0.751)	1.726 (0.330)	0.811 (0.459)
ARB (yes = 1)	-0.062 (0.910)	**1.066 (0.030)**	-17.380 (0.592)	1.174 (0.642)	0.484 (0.385)	1.691 (0.410)	1.570 (0.157)
Statin (yes = 1)	-0.225 (0.602)	0.404 (0.309)	-3.567 (0.889)	**0.571 (0.029)**	0.773 (0.692)	1.112 (0.833)	0.970 (0.902)
Furosemide (yes = 1)	0.644 (0.178)	-0.395 (0.357)	-40.283 (0.154)	1.350 (0.642)	2.417 (0.239)	0.530 (0.258)	1.435 (0.187)
**Patients with RKF (*n* = 42)**							
Albumin (g/L)	-0.001 (0.989)	0.073 (0.205)	3.436 (0.299)	1.021 (0.584)	1.259 (0.050)	1.047 (0.508)	**1.017 (0.010)**
Beta blocker (yes = 1)	0.324 (0.466)	**0.648 (0.048)**	26.075 (0.168)	1.264 (0.261)	1.337 (0.832)	0.598 (0.190)	1.334 (0.265)
Calcium antagonist (yes = 1)	-0.003 (0.995)	0.145 (0.668)	-28.647 (0.133)	1.360 (0.132)	1.688 (0.322)	**0.432 (0.031)**	0.720 (0.209)
ACE-inhibitor (yes = 1)	-0.169 (0.707)	-0.517 (0.108)	7.709 (0.685)	0.775 (0.258)	0.741 (0.799)	1.097 (0.815)	0.822 (0.448)
ARB (yes = 1)	-0.075 (0.878)	0.453 (0.205)	-21.312 (0.307)	1.017 (0.938)	3.151 (0.072)	0.462 (0.069)	1.273 (0.398)
Statin (yes = 1)	0.346 (0.437)	0.048 (0.888)	3.586 (0.853)	0.894 (0.597)	1.170 (0.984)	0.900 (0.792)	0.644 (0.088)
Furosemide (yes = 1)	0.173 (0.724)	**-0.856 (0.008)**	5.689 (0.788)	0.793 (0.358)	1.486 (0.437)	0.910 (0.829)	0.937 (0.822)

ACE-inhibitor, angiotensin-converting enzyme inhibitor; ARB, angiotensin II receptor blocker; CVD, cardiovascular disease; HsCRP, high-sensitivity C-reactive protein; nPNA, normalized protein nitrogen appearance. *Multivariate linear regression analysis with adjustment for age, gender and renal creatinine clearance. Data are expressed as the unstandardized beta regression coefficient (β) with *p*-value. The plasma concentration of kynurenine, kynurenic acid and indoxyl sulfate will increase with β, and the plasma concentration of hippuric acid, p-Cresyl glucuronide and indole-3-acetic acid will be multiplied by e^{β} if the variable increases by 1 unit or if the variable changes from 0 to 1 for a dichotomous variable. †Ordinal logistic regression analysis with adjustment for age, gender and creatinine clearance for p-Cresyl sulfate tertiles (tertile 1: <337 μmol/L, tertile 2: 337-694 μmol/L, tertile 3: >694 μmol/L). Data are expressed

as e^{β} with *p*-value which denotes the odds of having a plasma concentration in a higher tertile if the variable increases by 1 unit or changes from 0 to 1 for a dichotomous variable. Significant associations are shown in bold font.

Table S4. Hazard ratios for all-cause mortality and new cardiovascular events for PBUT tertiles at baseline.

				Hazard ratio (95% CI)			
**PBUT tertiles***	**Outcome**	**N**	**# Events**	**Model I**	***P***	**Model II**	***P***
Kynurenine	All-cause mortality	79	34	1.105 (0.736 to 1.658)	0.631	0.943 (0.615 to 1.445)	0.786
	CV events	78	29	1.172 (0.750 to 1.833)	0.486	1.070 (0.637 to 1.797)	0.797
Kynurenic acid	All-cause mortality	80	35	0.754 (0.490 to 1.160)	0.200	0.720 (0.399 to 1.297)	0.274
	CV events	79	29	0.861 (0.535 to 1.384)	0.536	1,492 (0.739 to 3.014)	0.265
Indoxyl sulfate	All-cause mortality	80	35	0.890 (0.582 to 1.360)	0.590	0.950 (0.541 to 1.669)	0.858
	CV events	79	29	1.097 (0.692 to 1.738)	0.693	1.510 (0.761 to 2.995)	0.239
Indole-3-acetic acid	All-cause mortality	60	24	1.146 (0.690 to 1.904)	0.599	1.220 (0.623 to 2.389)	0.563
	CV events	59	20	1.360 (0.789 to 2.344)	0.268	2.304 (0.980 to 5.416)	0.056
p-Cresyl sulfate	All-cause mortality	80	35	1.107 (0.732 to 1.674)	0.629	1.034 (0.643 to 1.663)	0.891
	CV events	79	29	0.886 (0.567 to 1.386)	0.597	0.844 (0.491 to 1.451)	0.540
p-Cresyl glucuronide	All-cause mortality	80	35	1.137 (0.739 to 1.749)	0.560	1.044 (0.653 to 1.669)	0.858
	CV events	79	29	1.035 (0.651 to 1.645)	0.883	1.235 (0.738 to 2.067)	0.421
Hippuric acid	All-cause mortality	78	33	0.950 (0.621 to 1.456)	0.815	0.895 (0.542 to 1.478)	0.665
	CV events	77	28	0.881 (0.554 to 1.401)	0.592	1.023 (0.564 to 1.855)	0.940

Uni/Multivariate Cox proportional hazards regression analysis censored for kidney transplantation. Model I: univariate; model II: adjustment for age, gender, diabetes mellitus, history of cardiovascular disease, renal creatinine clearance and normalized protein nitrogen appearance. Data are expressed as hazard ratios with 95% confidence interval (CI) and *p*-value for the occurrence of new cardiovascular (CV) events and all-cause mortality associated with a PBUT plasma concentration in a higher tertile. *PBUT plasma concentrations were divided into tertiles of kynurenine (<3.6, 3.6–4.9 and >4.9 µmol/L), kynurenic acid (<1.5, 1.5–2.5 and >2.5 µmol/L), indoxyl sulfate (<123, 123–180 and >180 µmol/L), indole-3-acetic acid (<5.9,

5.9–9.2 and >9.2 µmol/L), p-cresyl sulfate (<337, <337–701 and >701 µmol/L), p-cresyl glucuronide (<5.1, 5.1–20.5 and >20.5 µmol/L), and hippuric acid (<141, 141–273 and >273 µmol/L).

Table S5. Hazard ratios for all-cause mortality and new cardiovascular events for PBUT plasma concentrations at follow up.

				Hazard ratio (95% CI)			
**PBUT**	**Outcome**	**N**	**# Events**	**Model I**	***P***	**Model II**	***P***
Kynurenine (µmol/L)	All-cause mortality	74	33	0.891 (0.653 to 1.215)	0.464	0.822 (0.553 to 1.222)	0.322
	CV events	74	27	0.866 (0.626 to 1.199)	0.387	0.735 (0.456 to 1.185)	0.206
Kynurenic acid (µmol/L)	All-cause mortality	74	33	0.854 (0.628 to 1.162)	0.315	0.867 (0.527 to 1.426)	0.574
	CV events	74	27	0.713 (0.495 to 1.026)	0.068	0.710 (0.414 to 1.218)	0.214
Indoxyl sulfate (µmol/L)	All-cause mortality	74	33	1.003 (0.999 to 1.007)	0.143	1.003 (0.997 to 1.008)	0.309
	CV events	74	27	1.000 (0.995 to 1.005)	0.964	0.998 (0.991 to 1.005)	0.525
Indole-3-acetic acid (µmol/L)	All-cause mortality	52	22	0.844 (0.408 to 1.744)	0.647	0.907 (0.406 to 2.027)	0.812
	CV events	52	21	1.033 (0.524 to 2.034)	0.926	1.017 (0.492 to 2.102)	0.964
p-Cresyl sulfate (µmol/L)	All-cause mortality	74	33	0.952 (0.709 to 1.278)	0.742	0.905 (0.659 to 1.245)	0.540
	CV events	74	27	0.873 (0.650 to 1.174)	0.369	0.850 (0.595 to 1.213)	0.370
p-Cresyl glucuronide (µmol/L)	All-cause mortality	74	33	1.017 (0.817 to 1.268)	0.878	1.048 (0.835 to 1.314)	0.686
	CV events	74	27	0.941 (0.759 to 1.167)	0.580	1.035 (0.818 to 1.310)	0.776
Hippuric acid (µmol/L)	All-cause mortality	74	33	1.219 (0.858 to 1.733)	0.268	1.107 (0.713 to 1.720)	0.651
	CV events	74	27	0.761 (0.522 to 1.109)	0.155	0.855 (0.522 to 1.400)	0.533

Uni/Multivariate Cox proportional hazards regression analysis censored for kidney transplantation. Model I: univariate; model II: adjustment for age, gender, diabetes mellitus, history of cardiovascular disease, renal creatinine clearance and normalized protein nitrogen appearance. Data are expressed as hazard ratios with 95% confidence interval (CI) and *p*-value for the occurrence of new cardiovascular (CV) events and all-cause mortality associated with a one-unit increase in PBUT plasma concentration.

Table S6. Hazard ratios for all-cause mortality and new cardiovascular events for PBUT tertiles at follow up.

				Hazard ratio (95% CI)			
**PBUT tertiles***	**Outcome**	**N**	**# Events**	**Model I**	***P***	**Model II**	***P***
Kynurenine	All-cause mortality	74	36	0.756 (0.488 to 1.173)	0.212	0.687 (0.412 to 1.147)	0.151
	CV events	74	30	0.735 (0.461 to 1.173)	0.197	0.557 (0.312 to 0.995)	0.048
Kynurenic acid	All-cause mortality	74	36	0.686 (0.441 to 1.065)	0.093	0.607 (0.333 to 1.106)	0.103
	CV events	74	30	0.619 (0.377 to 1.016)	0.058	0.674 (0.345 to 1.316)	0.248
Indoxyl sulfate	All-cause mortality	74	36	1.337 (0.878 to 2.037)	0.176	1.281 (0.726 to 2.261)	0.393
	CV events	74	30	1.373 (0.856 to 2.202)	0.188	1.408 (0.671 to 2.958)	0.366
Indole-3-acetic acid	All-cause mortality	52	24	0.874 (0.517 to 1.475)	0.613	0.874 (0.480 to 1.589)	0.658
	CV events	52	24	1.048 (0.616 to 1.782)	0.863	1.236 (0.637 to 2.398)	0.530
p-Cresyl sulfate	All-cause mortality	74	36	0.986 (0.643 to 1.512)	0.949	0.861 (0.548 to 1.352)	0.515
	CV events	74	30	0.659 (0.409 to 1.060)	0.086	0.552 (0.326 to 0.935)	**0.027**
p-Cresyl glucuronide	All-cause mortality	74	36	0.992 (0.636 to 1.546)	0.972	1.092 (0.656 to 1.815)	0.736
	CV events	74	30	0.790 (0.488 to 1.277)	0.336	0.925 (0.527 to 1.625)	0.787
Hippuric acid	All-cause mortality	74	36	1.102 (0.729 to 1.665)	0.646	0.919 (0.585 to 1.445)	0.715
	CV events	74	30	0.678 (0.423 to 1.086)	0.106	0.708 (0.413 to 1.215)	0.210

Uni/Multivariate Cox proportional hazards regression analysis censored for kidney transplantation. Model I: univariate; model II: adjustment for age, gender, diabetes mellitus, history of cardiovascular disease, renal creatinine clearance and and normalized protein nitrogen appearance. Data are expressed as hazard ratios with 95% confidence interval (CI) and *p*-value for the occurrence of new cardiovascular (CV) events and all-cause mortality associated with a PBUT plasma concentration in a higher tertile. Significant differences are shown in bold font.*PBUT plasma concentrations were divided into tertiles of kynurenine (<3.5, 3.5–4.5 and >4.5 µmol/L), kynurenic acid (<1.7, 1.7–2.9 and >2.9 µmol/L), indoxyl sulfate (<115, 115–196 and >196 µmol/L), indole-3-acetic acid (<5.8, 5.8–9.0 and >9.0 µmol/L), p-cresyl sulfate (<336, <336–626 and >626 µmol/L), p-cresyl glucuronide (<5.3, 5.3–17.9 and >17.9 µmol/L), and hippuric acid (<101, 101–283 and >283 µmol/L).

Table S7. Hazard ratios for all-cause mortality and new cardiovascular events for the percentage change in PBUT plasma concentrations from baseline to follow up.

**Δ PBUT (%change/6 months)**	**Outcome**	**N**	**# Events**	**Hazard ratio (95% CI)**			
				**Model I**	***P***	**Model II**	***P***
Kynurenine	All-cause mortality	41	32	0.991 (0.980 to 1.002)	0.104	0.992 (0.980 to 1.005)	0.225
	CV events	73	27	0.990 (0.978 to .002)	0.107	0.987 (0.971 to 1.003)	0.098
Kynurenic acid	All-cause mortality	74	33	0.996 (0.988 to 1.005)	0.402	0.996 (0.987 to 1.006)	0.484
	CV events	74	27	0.992 (0.982 to 1.002)	0.102	0.988 (0.976 to 1.001)	0.061
Indoxyl sulfate	All-cause mortality	74	33	0.999 (0.992 to 1.007)	0.835	0.998 (0.988 to 1.007)	0.623
	CV events	74	27	0.991 (0.982 to 1.002)	0.065	0.983 (0.971 to 0.996)	**0.009**
Indole-3-acetic acid	All-cause mortality	43	17	0.996 (0.987 to 1.005)	0.377	0.995 (0.983 to 1.007)	0.409
	CV events	43	16	1.001 (0.995 to 1.008)	0.683	0.999 (0.993 to 1.006)	0.842
p-Cresyl sulfate	All-cause mortality	74	33	0.999 (0.995 to 1.003)	0.608	0.998 (0.990 to 1.006)	0.627
	CV events	74	27	0.999 (0.993 to 1.003)	0.458	0.992 (0.983 to 1.002)	0.134
p-Cresyl glucuronide	All-cause mortality	74	33	1.000 (0.999 to 1.001)	0.978	1.001 (0.998 to 1.004)	0.385
	CV events	74	27	1.000 (0.998 to 1.001)	0.732	0.999 (0.995 to 1.003)	0.606
Hippuric acid	All-cause mortality	73	32	0.999 (0.996 to 1.003)	0.709	0.999 (0.994 to 1.003)	0.565
	CV events	73	27	0.999 (0.995 to 1.003)	0.491	0.999 (0.994 to 1.004)	0.693

Uni/Multivariate Cox proportional hazards regression analysis censored for kidney transplantation. Model I: univariate; model II: adjustment for age, gender, diabetes mellitus, history of cardiovascular disease, renal creatinine clearance and normalized protein nitrogen appearance. Data are expressed as hazard ratios with 95% confidence interval (CI) and *p*-value for the occurrence of new cardiovascular (CV) events and all-cause mortality associated with an increase in the percentage change in PBUT plasma concentration from baseline to six months (Δ PBUT, %change/6 months). Significant differences are shown in bold font.
